# The bidirectional relationship between destructive leadership and organizational commitment

**DOI:** 10.3389/fpsyg.2025.1653061

**Published:** 2025-09-22

**Authors:** Jorge A. Elgueta, Martin Grill

**Affiliations:** Department of Psychology, University of Gothenburg, Gothenburg, Sweden

**Keywords:** destructive leadership, organizational commitment, job demands-resources model, self-determination theory, longitudinal studies, latent growth-curve modeling, industrial psychology, psychosocial work environment

## Abstract

**Introduction:**

This study aimed to determine how destructive leadership and organizational commitment relate to each other across time.

**Methods:**

Over 18 months, a self-rated questionnaire was distributed at four timepoints to employees of local government organizations in a municipality in Sweden; 582 employees responded to the questionnaire on at least one occasion. The Destructive Leadership Scale was used to measure destructive leadership, and the Commitment to the Workplace scale was used to measure organizational commitment. A second-order parallel-process latent growth curve model was used to test if initial levels of destructive leadership predict later change in organizational commitment, if initial levels of organizational commitment predict later change in destructive leadership, and if changes in destructive leadership are associated with simultaneous changes in organizational commitment.

**Results:**

The results show that the initial levels of destructive leadership negatively predicted change in organizational commitment (*β* = −0.66, *p* < 0.001), the initial levels of organizational commitment negatively predicted change in destructive leadership (*β* = −0.84, *p* < 0.002), and the rate of change in destructive leadership was strongly and negatively associated with the rate of change in organizational commitment (*r* = −0.96, *p* < 0.001).

**Discussion:**

The findings indicate that destructive leadership affects the work environment in a negative way by undermining employee’s organizational commitment. At the same time, managers are negatively affected by uncommitted employees, exacerbating destructive leadership behaviors.

## 1 Introduction

Over the past decades, research has shown how leadership plays a crucial role in organizations, affecting multiple outcomes. Empirical evidence suggests that effective leadership is positively associated with employees job satisfaction and well-being (An et al., 2020; Dalgaard et al., 2025; Fernemark et al., 2024; Shanafelt et al., 2015; Skakon et al., 2010) as well as decreased turnover intention, absenteeism, stress, and burnout (Dalgaard et al., 2025; Martinussen et al., 2020; Nyberg et al., 2008; Skakon et al., 2010). Leadership styles and their associated behaviors constitute important elements affecting both job demands and resources (Rasak et al., 2024), which have been used to explain the relationship between leadership and variables such as organizational commitment, job satisfaction, and work engagement (Atiku and Van Wyk, 2024; Claes et al., 2023; Rasak et al., 2024).

The existing literature on leadership shows three important limitations: Firstly, research tends to focus on the benign influence of effective leadership but often neglects the “dark side” of leadership. Secondly, the relationships are assumed to be unidirectional, with research only focusing on the effect of leadership on psychosocial and productivity variables; much less research has been devoted to investigating how psychosocial and productivity variables influence leadership styles and behaviors. Lastly, most of the research is cross-sectional, which limits the ability to make causal claims. To build upon the described relationships between leadership and employee outcomes, it is necessary to consider how ineffective leadership causally relates to psychosocial variables, such as organizational commitment, by using longitudinal data. In sum, the present study aims to address a gap by examining the negative effects of destructive leadership while also considering the bidirectional nature of the leader-employee dynamics across time.

Perhaps the most well-known leadership theory is the full-range leadership model (FRLM), which posits that leadership styles and behaviors vary in terms of their effectiveness and whether a leader exhibits passive or active behaviors (Avolio, 2010; Bass and Avolio, 1994). Originally, three broad leadership styles were described, one of them being repeatedly labeled “destructive” (Buch et al., 2015, p. 115; Klasmeier et al., 2022, p. 406; Skogstad et al., 2007, p. 80); laissez-faire leadership, often described as non-leadership, is characterized by high ineffectiveness (i.e., failing to accomplish shared objectives) and mainly consists of passive behaviors (Avolio, 2010). Empirically, laissez-faire leadership has been negatively correlated with organizational commitment, job satisfaction, workplace belonging, and recognition (Dumdum et al., 2013; Emirie and Mengistu Gebremeskel, 2024; Judge and Piccolo, 2004; Salamon et al., 2024) and positively correlated with role conflict and role ambiguity (Skogstad et al., 2007). Additionally, abusive supervision has been described as a leadership style characterized by hostile treatment of subordinates, directly undermining the accomplishment of organizational and individual goals. An abusive leader often exhibits hostile behaviors toward employees (such as angry outbursts, ridiculing, taking credit for their work, etc.) and impacts negatively on employees’ job satisfaction, meaning of work, organizational commitment, and job performance (Caesens et al., 2019; Griffin and Lopez, 2005; Harris et al., 2007; Mackey et al., 2017; Tepper, 2007).

The original full-range leadership model only considered two dimensions among which leadership styles vary; that is, passive – active, and ineffective – effective. Abusive supervision suggests another dimension to consider when describing leadership: hostile – nonhostile. Laissez-fare leadership can be characterized as being passive, ineffective, and non-hostile, while abusive supervision can be described as active, ineffective, and hostile. However, a considerable proportion of managers’ leadership behaviors is active, ineffective, and nonhostile. Until recently, such leadership has not been appreciated and scrutinized within the literature (Grill, 2023).

Grill (2023) described destructive leadership as being characterized by four active and ineffective but nonhostile behaviors, which negatively affect employees’ meaning of work and productivity. The first of these behaviors is incoherent planning, which reduces predictability and undermines employees’ long-term planning by forcing them to react to their manager’s changes in planning or direction. The second active but non-hostile destructive behavior leaders engage in is assigning unnecessary tasks, which creates additional demands for employees and is often perceived as disrespectful. The third destructive leadership behavior is communicating ambiguous expectations, which reduces role clarity and makes it hard for employees to understand how they are contributing to organizational goals. Lastly, the fourth behavior described is ignoring others’ views (labeled “autocratic behavior”), which can be perceived as devaluing and leads to employees being discouraged from actively participating in work-related activities.

In recent years, personnel turnover has become one of the main concerns for organizations globally, as high rates of voluntary turnover have become a growing problem; at an organizational level, a high rate of turnover incurs in higher financial costs associated with recruitment and training, as well as decreases in productivity (Cregård et al., 2017; Eurostat, 2023; Gallup, 2025; Staten i siffror: personalomsättning, 2025). The organizational literature outlines a few major predictors of turnover, one of the main ones being organizational commitment (Hur and Abner, 2024; Meyer et al., 2002; Westerberg et al., 2021).

Commitment is also at the core of what in recent years has been popularized as “quiet quitting,” which refers to when employees fulfil their duties “only to the minimal extent required (not to get fired)”; “negative leadership” is highlighted as one of the factors driving quiet quitting, and organizations affected by this phenomenon incur in losses of productivity, innovation capacity, and long-term competitiveness (Kim and Sohn, 2024, pp. 459–460). Understanding how leadership may affect organizational commitment is of paramount importance considering these challenges. Researchers have defined organizational commitment as the strength of an individual’s identification and involvement with an organization (Meyer and Allen, 1991; Porter et al., 1974). Leader consideration, role ambiguity, role conflict, and the influence employees can exert over their own work (i.e., autonomy) have been proposed as important antecedents of organizational commitment that are directly influenced by leadership (Bodjrenou et al., 2019; Cohen, 1993; Corin, 2016; Mathieu and Zajac, 1990).

According to the job demands-resources model (JD-R), job demands refer to the psychological, cognitive, social, or organizational aspects of a job that require sustained effort and incur in physiological and/or psychological costs. On the other hand, job resources are thought of as the aspects of a job that reduce the effect of job demands and are functional toward achieving organizational goals (Bakker and Demerouti, 2017). The JD-R model provides a theoretical framework through which we can understand how high job demands, when coupled with low resources result in a diminished organizational commitment and, ultimately, a diminished job performance (Priyono et al., 2022; Sørlie et al., 2022). This is also consistent with the broader Conservation of Resources (COR) theory, which holds that people in general strive to acquire and protect valuable resources, with stress and burnout being the result of either perceived or actual threats to these resources (Hobfoll, 1989; Hobfoll et al., 2018).

In addition, the Self-Determination Theory (SDT) provides possible explanations for how destructive leadership could influence organizational commitment by diminishing employees’ resources. SDT highlights competence, relatedness, and autonomy as fundamental human needs. Autonomy is often highlighted as a decisive factor in intrinsic motivation and commitment (Ryan and Deci, 2000). In organizational settings, it has been suggested that the interpersonal style of leaders may be an important factor that influences job demands and resources (Gagné and Deci, 2005; Olafsen and Deci, 2020). Consequently, it can be argued that the four distinctive behaviors of destructive leadership may erode job resources for employees, ultimately undermining their autonomy.

For the current study, we find the JD-R to be the more appropriate framework, as it allows for more specific hypotheses, is specifically tailored to organizational settings, and provides with rich empirical backing for the aims of the current research. Within this framework, it has been pointed out that leadership simultaneously influences both job demands and resources. For example, laissez-faire leadership has been negatively linked to perceived support, fairness, and organizational commitment, as it limits employees’ resources (Jackson et al, 2013; Zheng and Li, 2024). In other words, based on the JD-R model, the impact of leadership on organizational commitment could be linked to the managers’ influence over employees’ job demands and resources; destructive leadership may exacerbate job demands through incoherent planning and the assignment of unnecessary tasks, while also diminishing employee’s job resources, such as influence over their own work, through autocratic behavior (i.e., ignoring others’ views) (Grill, 2023).

Based on the JD-R model, SDT, and the empirical evidence available, the initial hypothesis for the present study is:

*H1*: Destructive leadership predicts diminishing organizational commitment.

Just as their subordinates, leaders are exposed to differing job demands and resources, which in turn may affect their health, motivation, and performance (Tóth-Király et al., 2024). Particularly, managers working for government organizations may be more exposed to contextual factors that heighten demands while limiting resources, such as role ambiguity and conflict resulting from contradictory objectives; one example of conflicting objectives is the demand to operate with differing budgets based on political considerations and priorities, while maintaining quality (Berntson et al., 2012; Corin, 2016). However, another important aspect that managers face is having to deal with conflicts with subordinates, conflicts among subordinates, and unmotivated employees; not being able to trust subordinates and managing employees that are unwilling to do their job is often part of the demands inherent to a leadership position (Berntson et al., 2012; Corin, 2016). When interpreting these findings through the JD-R model, one could make the argument that operating with a limited budget and having to manage interpersonal conflict at the workplace represent heightened demands on public-sector managers. On the other hand, not being able to trust employees, dealing with unmotivated subordinates, and a limited autonomy based on policy direction and regulatory frameworks represent diminished resources. Lastly, uncommitted employees, who are often less productive and more likely to quit their job (Hur and Abner, 2024; Kim and Sohn, 2024; Meyer et al., 2002; Westerberg et al., 2021), represent a further drain on financial resources associated with diminished productivity and recruiting costs. At the same time, managers are required to provide results even on strained budgets and understaffed teams.

The imbalance between job demands and resources has been studied as a direct antecedent to negative health outcomes such as burnout, stress, anxiety and sleep problems, which represent a further reduction in personal resources (Bakker et al., 2007, 2005; Demerouti et al., 2001; Xanthopoulou et al., 2007). High and unbalanced job demands also affect work performance negatively (Nahrgang et al., 2011), with overwhelmed managers often entering a feedback loop in which stress and burnout lead to poorer performance, which in turn results in more stress. An important aspect of managers’ performance is their ability to lead their teams in an effective manner through sound decision-making, clear communication, and adequate support. However, in contexts of high stress and burnout, managers performance is affected both cognitively and behaviorally. Cognitively, stress has been found to be associated with impaired decision-making, concentration, and impulse control (Chrusciel et al., 2024; Corin, 2016). On the other hand, overwhelmed managers may withdraw and become more cynical (i.e., displaying a distant attitude toward work in general), becoming less available to their team and failing to provide guidance to their employees and thereby reducing their productivity (Bakker et al., 2014; Corin, 2016). Based on these findings, it is conceivable that, through an impaired decision-making process, a manager may end up planning ineffectively and creating unnecessary tasks for subordinates. Furthermore, the drain on cognitive resources may also result in autocratic behaviors (i.e., ignoring others’ views) to reduce the uncertainty and complexity inherently associated with decision-making (Barnes et al., 2011; Fiedler and Garcia, 1987). It could also be the case that a behaviorally withdrawn and cynical manager may be perceived as autocratic, disregarding others’ views or not bothering to request them in the first place. Lastly, a withdrawn manager may end up communicating their expectations in an ambiguous manner. Following these observations, the second hypothesis of the current study is:

*H2*: Lower organizational commitment predicts increasing destructive leadership.

The first two hypotheses in the present study are designed to capture the unique direct effects of destructive leadership on change in organizational commitment and of organizational commitment on change in destructive leadership. However, this relationship may be more dynamic, as neither destructive leadership behaviors nor organizational commitment are stable over time. On the contrary, changes in destructive leadership could be associated with simultaneous changes in organizational commitment. For example, Manville et al. (2023) found that subordinates’ perception of abusive supervision fluctuates over time, owing to variations in supervisor’s resource depletion, lapses or improvements on performance, and achievements. Similarly, Diebig and Bormann (2020) found that daily change in laissez-faire leadership tracks closely with daily change in perceived stress of employees. In line with our arguments in the previous section, note that the variability in leadership behaviors may be due to leaders being limited in their capacity to lead effectively due to depleted personal and organizational resources, as well as increases in job demands.

As for organizational commitment, Maia et al. (2016) found that positive change in role overload, defined as when an individual experiences a lack of resources needed to fulfil their duties, is a strong predictor of negative change in commitment. Additionally, employee’s unmet expectations are widely cited as another factor that diminishes employees’ organizational commitment (Bentein, 2016; Chen et al., 2008; Robinson and Wolfe Morrison, 2000). Hence, employee commitment may be one of the resources that facilitates managerial work and, when commitment deteriorates, this resource is reduced. On the other hand, when managers assign unnecessary tasks, demonstrate deficient planning, communicate unclear expectations, and engage on autocratic behaviors, they exacerbate work demands and fail to live up to their employees’ expectations. Consequently, we hypothesize that:

*H3*: There is a negative association between change in destructive leadership and change in organizational commitment.

## 2 Materials and methods

### 2.1 Procedure and participants

The data for the current study was originally collected by Grill (2023) from employees (*n* = 712) in local government organizations in a municipality in Sweden. The operations pertaining to these organizations included education, healthcare, construction and real-estate development, management and maintenance of property and infrastructure, social and emergency services, elderly care, transport, administration and economics, cultural administration, tourism, communication, and human resource management. Data was collected via an online questionnaire, which was sent out to employees at four timepoints, separated by six-month intervals: October–November 2019 (T1), April–May 2020 (T2), October–November 2020 (T3), and April–May 2021 (T4). Updated e-mail lists were used at each timepoint to allow for the inclusion of recently employed individuals. 586 employees (82.3%) answered the questionnaire at least once. Respondents were 57% female, with an average age of 45 years (SD = 11.85), and 73.7% had a university education. Table 1 shows demographics for the sample of respondents at each timepoint. Employees were clustered under 72 managers, who had an average age of 47 years old (SD = 6.8), with 57% being female.

**Table 1:** Descriptive statistics for the sample at each timepoint.

Time-point	*N*	Mean age	Sd	Female	College-educated
T1	498	44.21	11.44	53.83%	70.88%
T2	447	44.89	12.13	57.49%	72.70%
T3	369	45.19	12.09	59.07%	77.50%
T4	297	45.74	11.74	57.48%	73.73%

### 2.2 Materials

Destructive Leadership (DL) was measured at all timepoints using the four-item Destructive Leadership Scale developed by Grill (2023), which includes 4 types of active, non-hostile, and destructive leadership behaviors: incoherent planning (“How often does your manager demonstrate deficient planning behaviors?”), assigning unnecessary tasks (“How often does your manager make decisions that generate unnecessary tasks?”), ambiguous expectations (How often does your manager express ambiguous expectations?”), and autocratic behavior (“How often does your manager ignore your views?”). Responses were recorded on a Likert-type scale ranging from 1 (never) to 5 (frequently, if not always).

Organizational Commitment (CW) was measured using the Commitment to the workplace scale, developed for the Copenhagen Psychosocial Questionnaire, second version (COPSOQ II) (Pejtersen et al., 2010). The scale measure three items; that is, “Do you feel that your place of work is of great importance to you?”, “Would you recommend other people to apply for a position at your workplace?”, and “How often do you consider looking for work elsewhere?” The items are rated on a Likert-type scale from 1 to 5 (for the first two items, the scale range from “to a very low degree” to “to a very high degree”; for the last item, the scale range from “always” to “never/almost never”).

For DL, McDonald’s *ω* ranged from 0.80 to 0.83 across timepoints, indicating adequate reliability. The instrument showed low-to-moderate clustering effects, with the intra-class correlation coefficient (ICC) ranging from 0.10 to 0.23. DL showed adequate convergent validity, with standardized factor loadings ranging between 0.65 and 0.82. When dealing with clustered data, multi-level modeling is usually recommended (Grimm et al., 2017). However, the low-to-moderate ICC values indicate that most of the variance was located on the individual level. Following these considerations, clustering effects were accounted for by using a robust maximum likelihood estimator (MLR) for all subsequent models. MLR provides comparatively unbiased standard errors and *p*-values, is less sensitive to non-normality, and allows for more accurate inferences about the significance of the models (Chou et al., 1991). After running a single-factor solution CFA on DL for all timepoints, fit indices at T1, T2 & T3 showed excellent fit, as judged by CFI > 0.95, TLI > 0.95, and SRMR < 0.08. At T4, the TLI (0.74) indicated potential misfit. Additionally, the RMSEA for two timepoints (T2 = 0.13; T4 = 0.27) surpassed the conventional threshold of 0.08. The low TLI at T4 and the elevated RMSEA at T2 and T4 could hint at the construct not being unidimensional across time-points, possibly compromising measurement invariance (this issue was further analyzed in the invariance tests below).

For CW, McDonald’s ω ranged from 0.75 to 0.81 across timepoints, indicating adequate reliability (fit indices for a single-factor CFA are uninformative due to the model being just-identified). The scale for CW also exhibited low-to-moderate ICC values, which ranged from 0.08 to 0.19. CW showed acceptable convergent validity, with standardized factor loadings ranging between 0.59 and 0.78. Discriminant validity was assessed by using the Fornell-Larcker criterion, which requires the square root of each construct’s Average Variance Extracted (√AVE) to exceed its correlation with other constructs (Fornell and Larcker, 1981). The √AVE ranged between 0.73 and 0.77 for DL and between 0.67 and 0.77 for CW, with √AVE values being greater than the correlations observed between constructs. Detailed descriptive statistics for the study variables–including reliability measures, clustering effects, means, variance, √AVE, and intercorrelations–are shown in Table 2.

**Table 2:** Descriptive statistics for study variables.

Variable	Time-point	Mean	σ^2^	√AVE	Reliability	Clustering	Intercorrelations							
					ω	ICC	1	2	3	4	5	6	7	8
DL	T1	2.18	0.57	0.73	0.80	0.10	1							
	T2	2.15	0.63	0.74	0.84	0.15	0.79*	1						
	T3	2.23	0.63	0.74	0.82	0.14	0.78*	0.78*	1					
	T4	2.26	0.72	0.76	0.83	0.23	0.64*	0.70*	0.69*	1				
CW	T1	3.65	0.39	0.68	0.75	0.08	−0.54*	−0.46*	−0.47*	−0.30*	1			
	T2	3.70	0.36	0.67	0.74	0.19	−0.53*	−0.64*	−0.59*	−0.40*	0.85*	1		
	T3	3.63	0.42	0.70	0.77	0.15	−0.47*	−0.49*	−0.64*	−0.47*	0.75*	0.86*	1	
	T4	3.56	0.56	0.74	0.81	0.19	−0.45*	−0.50*	−0.51*	−0.70*	0.64*	0.69*	0.78*	1

*n* = 561; ICC was calculated using a two-way mixed-effects model; A confirmatory factor analysis—with the intercepts constrained to zero and the factor loadings to one for the first item in each scale—was used to provide descriptive statistics and correlations between the study variables [*χ*^2^ (336) = 861.30, *n* = 586, CFI = 0.937, TLI = 0.929, RMSEA = 0.061, 95% RMSEA [0.056–0.066], SRMS = 0.138]; Fit indices are robust; DL, destructive leadership; CW, organizational commitment; σ^2^, variance; *p < 0.05.

To test for measurement invariance over time for both scales, and to further investigate the potential misfit observed for DL at T4, a series of CFAs were conducted, treating each timepoint as a latent variable (Widaman et al., 2010). Detailed fit indices for the invariance models are shown in Table 3. Error variances of corresponding items were allowed to covary across timepoints. DL held acceptable time invariance (ΔCFI < 0.01), indicating the destructive leadership construct to be measured consistently across timepoints (Chen, 2007). A similar approach was used for CW, which also showed time invariance across measurements.

**Table 3:** Invariance models.

Variable	Model	df	χ^2^	*p*	CFI	TLI	RMSEA	95% CI	SRMR
DL	Configural	75	162.70	< 0.001	0.977	0.964	0.05	0.04–0.07	0.14
	Metric	84	198.98	< 0.001	0.974	0.964	0.05	0.03–0.07	0.19
	Scalar	93	199.56	< 0.001	0.977	0.970	0.05	0.04–0.06	0.19
CW	Configural	30	75.29	< 0.001	0.989	0.975	0.06	0.04–0.07	0.13
	Metric	37	81.33	< 0.001	0.990	0.982	0.05	0.03–0.06	0.13
	Scalar	43	82.56	< 0.001	0.992	0.987	0.04	0.02–0.06	0.14

*n* = 561; Fit indices and means for univariate CFAs at each timepoint using MLR as estimator and FIML to handle missing values; Fit indices are robust; DL, destructive leadership; CW, organizational commitment.

### 2.3 Data analysis

The analyses were performed in R, version 4.4.2 (R Core Team, 2024), using the Lavaan package (Rosseel et al., 2024). Estimations were computed using MLR, and missing values were handled with full information maximum likelihood (FIML). All indicators were mean centered in order to mitigate potential collinearity. As a first step, two domain-specific second-order latent growth curves (LGCM) were modeled separately (i.e., univariate) for destructive leadership and organizational commitment to establish their mean trajectories and variability (Grimm et al., 2017). Corresponding indicators for both scales were allowed to covary across timepoints, and factor loadings and intercepts were constrained to be equal across time as justified by the strong (scalar) time invariance of the measurement models. Disturbance terms of the first-order factors were constrained to be equal across time, as they represent variability that is not accounted for at the second-order level (Grimm et al., 2017). The T1 loading for the slopes was constrained to zero, with the loadings for T2, T3, and T4 being constrained to one, two, and three, respectively.

**Figure 1:**
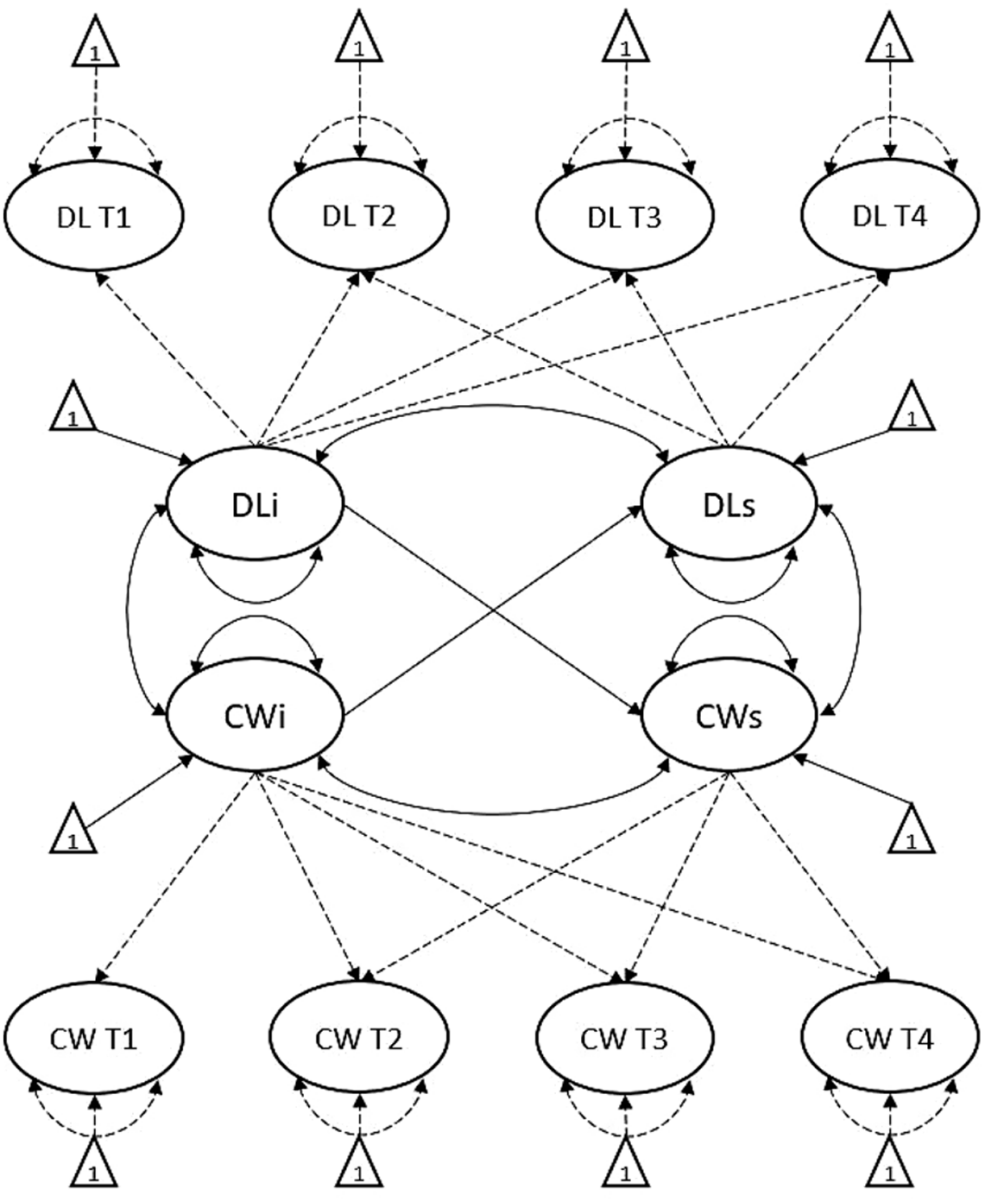
Dotted lines represent constrained paths; DL, destructive leadership; CW, organizational commitment; DLi, destructive leadership intercept; DLs, destructive leadership slope; CWi, organizational commitment intercept; CWs, organizational commitment slope.

In step two, second-order parallel process growth curves (PP-LGCM) were modeled to examine the relationships between the intercepts and slopes of destructive leadership and organizational commitment (Grimm et al., 2017). PP-LGCMs can be used to assess the relationships between growth factors of multiple variables simultaneously, therefore allowing to test whether the trajectory of destructive leadership tracks with the trajectory of organizational commitment and vice versa. The goal of the first model (Model 1) was to test the unique effect of the initial levels of destructive leadership on the subsequent rate of change in organizational commitment (H1), as well as the unique effect of the initial levels of organizational commitment on the subsequent rate of change in destructive leadership (H2). Corresponding intercepts and slopes were allowed to covary. The goal for the second model (Model 2) was to assess the covariance between the rate of change in destructive leadership and the rate of change in organizational commitment (H3). Initial levels of both variables were allowed to covary. The strong time-invariant latent variables modeled in the domain-specific LGCMs were also used for the PP-LGCMs. The path diagram for Model 2 is shown in Figure 1. Lastly, all analyses were rerun using listwise deletion (*n* = 219) to discard any undue bias caused by participants entering or leaving the study at different timepoints.

### 2.4 Ethics statement

The research involving humans were approved by the Swedish Ethical Review Authority, Dnr 1,060-18/2019-00590. The studies were conducted in accordance with the local legislation and institutional requirements. The participants provided their written informed consent to participate in this study.

## 3 Results

The results from the univariate LGCMs are shown in Table 4, which describe that initial levels of destructive leadership varied significantly among individuals (intercept σ^2^ = 0 0.418, *p* < 0.001). On average, destructive leadership increased slightly over time (slope *β* = 0.251, *p* = 0.035). The change in destructive leadership showed marginally significant individual variability (slope σ^2^ = 0 0.012, *p* = 0.076). Organizational commitment also showed individual variability in initial levels (intercept σ^2^ = 0 0.404, *p* = 0.001) and was decreasing slightly over time (slope *β* = −0.128, *p* = 0.105), although this change was non-significantly different from zero. Results showed significant individual variability in rates of change (slope σ^2^ = 0.026, *p* = 0.029).

**Table 4:** Univariate second-order latent growth curves.

Growth Factors	Destructive leadership					Organizational commitment				
	Estimate	SE	*z*	*p*	*β*	Estimate	SE	*z*	*p*	*β*
Intercept	0.00	–	–	–	0.00	0.00	–	–	–	0.00
σ^2^	0.418	0.047	8.808	< 0.001	1.00	0.404	0.122	3.306	0.001	1.00
Slope	0.027	0.013	2.107	0.035	0.251	−0.021	0.013	−1.623	0.105	−0.128
σ^2^	0.012	0.007	1.774	0.076	1.00	0.026	0.012	2.187	0.029	1.00

*n* = 561; destructive leadership (mean centered); organizational commitment (mean centered); intercepts were set to 0 for identification purposes; σ^2^, variance; destructive leadership robust fit, [*χ*^2^ (96) = 164.46, *n* = 561, CFI = 0.985, TLI = 0.981, RMSEA = 0.039, 95% RMSEA [0.028–0.049], SRMR = 0.049]; organizational commitment robust fit, [*χ*^2^ (46) = 66.92, *n* = 561, CFI = 0.996, TLI = 0.995, RMSEA = 0.025, 95% RMSEA [0.00–0.042], SRMR = 0.038].

Table 5 outlines the results of the PP-LGCMs. In the first model (Model 1), initial levels (i.e., the intercepts) of both variables were not significantly correlated to their later changes (i.e., their slopes). However, the unique direct effect of the intercept of destructive leadership on the slope of organizational commitment was negative and significant (*β* = −0.663, *p* < 0.001). In support of H1, this means that individuals with higher initial levels of destructive leadership tended to exhibit a steeper decline in organizational commitment over time. The unique direct effect of the intercept of organizational commitment on the slope of destructive leadership was also negative and significant (*β* = −0.838, *p* = 0.002). In line with H2, this finding shows that individuals with lower initial levels of organizational commitment exhibited a steeper increase in destructive leadership over time.

**Table 5:** Second-order parallel process growth curve models.

		Model 1					Model 2				
		Estimate	SE	*z*	*p*	Std.	Estimate	SE	*z*	*p*	Std.
Growth Factors of DL	Intercept	0.000	–	–	–	0.000	0.000	–	–	–	0.000
	σ^2^	0.338	0.075	4.534	<0.001	1.00	0.440	0.398	1.105	0.269	1.00
	Slope	0.027	0.013	2.022	0.043	0.428	0.030	0.021	1.416	0.157	0.282
	σ^2^	0.001	0.005	2.41	0.810	0.298	0.011	0.013	0.846	0.397	0.942
Growth Factors of CW	Intercept	0.000	–	–	–	0.000	0.000	–	–	–	0.000
	σ^2^	0.385	0.055	7.036	<0.001	1.00	0.479	0.213	2.247	0.025	1.00
	Slope	−0.028	0.013	−2.145	0.032	−0.169	−0.029	0.014	−2.31	0.042	−0.161
	σ^2^	0.016	0.004	3.497	<0.001	0.561	0.032	0.014	2.315	0.021	0.990
Paths	DLi ↔ DLs	0.014	0.014	0.968	0.333	0.690	0.008	0.017	0.499	0.618	0.123
	CWi ↔ CWs	−0.012	0.013	−0.973	0.331	−0.157	−0.023	0.015	−1.546	0.122	−0.189
	DLi → CWs	−0.191	0.030	−6.266	<0.001	−0.663	−0.027	0.041	−0.661	0.509	−0.100
	CWi → DLs	−0.086	0.028	−3.128	0.002	−0.838	0.037	0.042	0.876	0.381	0.241
	DLi ↔ CWi	–	–	–	–	–	−0.275	0.077	−3.591	<0.001	−0.600
	DLs ↔ CWs	–	–	–	–	–	−0.018	0.009	−2.049	0.040	−0.966

*n* = 561; DLi, destructive leadership intercept; DLs, destructive leadership slope; CWi, organizational commitment intercept; CWs, organizational commitment slope; σ^2^, variance; Model 1 robust fit [*χ*^2^ (328) = 676.16, *n* = 561, CFI = 0.957, TLI = 0.950, RMSEA = 0.051, 95% RMSEA [0.045–0.056], SRMR = 0.113]; Model 2 robust fit, [*χ*^2^ (326) = 595.90, *n* = 561, CFI = 0.968, TLI = 0.962, RMSEA = 0.044, 95% CI RMSEA [0.038–0.049], SRMR = 0.061].

In the second model (Model 2), which included the estimation of the covariance between the slope of destructive leadership and the slope of organizational commitment (as well as the covariance between the intercepts of both variables), the regression paths from the intercept of destructive leadership onto the slope of organizational commitment (*β* = −0.100, *p* = 0.509) and from the intercept of organizational commitment onto the slope of destructive leadership (*β* = 0.241, *p* = 0.381) became non-significant. Corresponding intercepts and slopes remained uncorrelated. However, initial levels of destructive leadership proved to be negatively correlated to initial levels of organizational commitment (*r* = −0.600, *p* < 0.001) and, in line with H3, we found a strong, negative correlation between the slopes of both constructs (*r* = −0.966, *p* = 0.040). This means that individuals with higher initial scores on destructive leadership tended have lower initial scores on organizational commitment, and individuals who experienced a steeper rise in destructive leadership also tended to experience a steeper decline in organizational commitment.

Sensitivity analyses using only participants with full data, *n* = 219, yielded largely similar results for the domain-specific LGCMs, as well as for both PP-LGCMs.

## 4 Discussion

The present study aimed to investigate the effects of destructive leadership on organizational commitment, the effects of organizational commitment on destructive leadership, and the reciprocal relationship between destructive leadership and organizational commitment over time. Consequently, we proposed three hypotheses: (H1) Destructive leadership predicts diminishing organizational commitment, (H2) Lower organizational commitment predicts increasing destructive leadership, and (H3) There is a negative association between change in destructive leadership and change in organizational commitment. To test these hypotheses, two second-order parallel process latent growth curves (PP-LGCM) were modeled.

In the first model (Model 1), the unique cross-variable effect of the intercept of destructive leadership on the change in organizational commitment proved to be strong, negative, and significant, providing support for H1; that is, destructive leadership results in lower organizational commitment. By some margin, H1 had the most empirical evidence to back it (Bodjrenou et al., 2019; Cohen, 1993; Corin, 2016; Mathieu and Zajac, 1990). Consistent with these studies, as well as with the Job Demands-Resources Model (JD-R) (Bakker and Demerouti, 2017), the decreasing trajectory observed for organizational commitment may be theoretically explained through the depleting influence of destructive leadership behaviors on employees’ resources; incoherent planning diminishes predictability, assigning unnecessary tasks reduces job autonomy, communicating unclear expectations reduces role clarity, and autocratic behavior diminishes emotional and instrumental support. Similarly, Grill (2023) found that destructive leadership reduces meaning of work and work productivity, with the effects accumulating over time. On the other hand, some destructive leadership behaviors also exacerbate job demands; incoherent planning demands that employees react to sudden shifts in direction, while assigning unnecessary tasks may result in tasks accumulating. The findings for H1 are also consistent with Self-Determination Theory (SDT) (Ryan and Deci, 2000), which explains the observed effect of destructive leadership behaviors on organizational commitment through undermining employees’ needs for competence, relatedness, and autonomy.

Model 1 also evidenced a large and negative cross-variable unique effect of the intercept of organizational commitment on the change in destructive leadership, which provides support for H2; low organizational commitment results in increasing destructive leadership behaviors. This finding is also consistent with the JD-R; commitment is an important predictor of increased voluntary turnover and quiet quitting (Hur and Abner, 2024; Jackson et al., 2013; Kim and Sohn, 2024; Westerberg et al., 2021), with both of these phenomena reducing productivity and innovation, and increasing expenditure. This could lead to a situation in which organizational departments are understaffed, experience increased strains on budget, and exhibit an overall low productivity. All of these elements can be understood as a depletion of managerial resources, as well as an increase of demands. This results in additional stress and burnout for managers, which might be the driving force behind the growth in destructive leadership behaviors; stress impairs decision-making, concentration, and dampens impulse control (Chrusciel et al., 2024; Corin, 2016), which may result in incoherent planning, communicating unclear demands, and assigning unnecessary tasks. On the other hand, burnout can also lead to managers withdrawing and displaying a distant attitude (Bakker et al., 2014; Corin, 2016), which is a plausible explanation for ignoring others’ views. H2 directly addresses one of the gaps in the literature and further establishes that managers’ leadership performance is affected by their employees (Larsman et al., 2023; Samuelsson et al., 2023) and highlights the relevance for further research into the influence of employee behavior on leadership behavior. Lastly, this finding, when interpreted in conjunction with H1, suggests that the relationship between the variables is bidirectional.

H3 also found ample support; that is, the strong negative correlation between the slopes of both constructs shows that, as individuals experience a steeper rise in destructive leadership, they also report steeper decline in organizational commitment. Although the initial levels of destructive leadership and organizational commitment were also negatively correlated, the relatively stronger correlation found between the rate of change for both variables suggest that the effects destructive leadership and organizational commitment have on each other may accumulate over time. This is in line with previous empirical evidence (Diebig and Bormann, 2020; Grill et al., 2024; Manville et al., 2023). In other words, fluctuations in the levels of one variable are reflected by the other, irrespective of initial levels. This underscores the highly dynamic nature of leadership and the psychosocial work environment and further reinforces the idea that the relationship is bidirectional.

Lastly, we offer a tentative explanation for why, in Model 2, the cross-variable unique effects of the initial levels in one variable on the rates of change in the other became non-significant. About 36% (*r*^2^ = 0.36) of the variance in the initial level of organizational commitment was explained by its relationship to the initial level of destructive leadership, while 93% (*r*^2^ = 0.93) of the variance in the slope of destructive leadership is explained by its relationship to the slope of organizational commitment. Such strong relationships subsume most of the residual variance that a regression model could otherwise explain, leaving little unique residual variance for the analyzed paths to account for. In other words, the covariance between initial levels and rates of change may have masked the causal relationships between the variables. Future research should address this by including control variables for both destructive leadership and organizational commitment.

### 4.1 Limitations and directions for future research

The main limitation of the present study is that, although it establishes both correlation and temporal precedence, it does not account for potential confounding variables. This limitation arises from the use of observational data, which precludes randomization and thus prevents ruling out alternative explanations for the relationships identified in the results. At an average of five participants per free parameter estimated, the study just about meets the minimum requirement of a 5:1 participants – free parameter ratio outlined by Bentler and Chou (1987), and falls short of the 10:1 ratio often cited as a rule-of-thumb recommendation for SEM (Bentler and Chou, 1987; Bollen, 1989; Kline, 2016; Tabachnick and Fidell, 2019). Therefore, to retain maximum statistical power, control variables were not included in the analysis. However, future studies should consider the influence of managers’ age, gender, and training on destructive leadership. For organizational commitment, important control variables to include would likely be job satisfaction and job tenure (Bodjrenou et al., 2019; Mathieu and Zajac, 1990; Meyer et al., 2002). The inclusion of control variables may allow for a more accurate estimation of the high correlations between intercepts and slopes, and consequently, allow for a better, more naturalistic assessment of the cross-variable regression paths. It is also important to mention that organizational commitment consists of different subdimensions that could be affected differently by destructive leadership behaviors. Measuring and modeling these in future studies would help to provide nuance to the results presented, as well as tailor intervention programs more specifically.

Additionally, variables pertaining to the structure of the organization should be considered, as they are also important when considering psychosocial variables. Vertical and bureaucratic structures, such as public organizations, are often limiting in terms of employee autonomy and participation (Ahmetoglu et al., 2019; Niveditha and Padhy, 2024). On the other hand, more horizontal organizations have been found to increase role ambiguity and conflict (Ebbers and Wijnberg, 2017; Nygaard and Dahlstrom, 2002). The Swedish context presents a particularly interesting challenge in this regard: while public organizations are commonly thought of as being more vertical, Swedish society is famous for its horizontality and its rule-by-consensus approach. Future research should aim to measure and control for the effect organizational structure variables on both leadership and psychosocial variables, as well as integrate more cultural-level variables that could affect managers’ and employees’ behaviors, such as individualism or distance to power.

A final limitation is that the data collection happened at the onset of the Covid-19 pandemic. This poses a challenge for generalization after such an important event, as both destructive leadership and organizational commitments could have been influenced by confounds outside the direct sphere of organizational psychology. Therefore, future replication of the study is needed in order to better assess the generalizability of the results presented.

## 5 Conclusion

The present research aimed to investigate the effects of destructive leadership on organizational commitment. Consistent with previous findings by Grill (2023), this study found that destructive leadership negatively impacts the work environment by undermining employee’s organizational commitment. These findings also align with the job demands-resources model and are particularly relevant, given that organizational commitment is a direct antecedent of turnover intention–a pressing issue in many organizations today.

The study also aimed to uncover how a low organizational commitment among employees might affect leadership behaviors. Findings suggest that managers may be negatively affected by uncommitted employees, exacerbating subtle, non-hostile but destructive leadership behaviors. Lastly, the study aimed to describe the dynamic relationship between destructive leadership and organizational commitment over time. Findings suggest that both constructs may be highly interactive and react to changes in the other. The results open interesting avenues for new research: most of the leadership literature has been devoted to understanding how managers can influence their employees and affect personal and organizational outcomes. However, there’s surprisingly little research about the inverse relationship. Our findings highlight that employee’s psychosocial characteristics, such as commitment, may constitute an important resource for managers. Without this resource, a manager may experience additional stress and burnout which, in turn, may be the driving force behind the heightened destructive leadership behaviors they engage in.

In sum, the value of the present study to the organizational literature is the integration of the job demands-resources model and self-determination theory to argue that destructive leadership and organizational commitment may be mutually reinforcing processes that evolve together, opening new perspectives within organizational psychology. By integrating the job demands-resources model with self-determination theory and using a longitudinal design, we highlight that organizational commitment is not only an outcome variable of leadership, but also an important resource that influences managerial behavior. This is a perspective that should be expanded in the future to include other psychosocial variables. It should also be considered when designing interventions, as findings suggest that that employee’s levels of commitment and other psychosocial variables may be important in understanding and influencing leadership behaviors.

## Data Availability

The raw data supporting the conclusions of this article will be made available by the authors, without undue reservation.
